# Supplementary material for: Does acute soccer heading cause an increase in plasma S100B? A randomized controlled trial
Source: PLoS One. 2020 Oct 23;15(10):e0239507. doi: 10.1371/journal.pone.0239507 (PMC7584162; doi:10.1371/journal.pone.0239507)
Supplement: S1 File — (DOCX) [file pone.0239507.s002.docx]

**Effects of soccer heading on ocular-motor function, brain-derived blood biomarker, and neuronal function**

## Keisuke Kawata, PhD, Assistant Professor

Kinesiology,

School of Public Health, Indiana University

1025 E. 7^th^ Street, SPH C215

Bloomington, IN 47401

**Hannah Block, PhD, Assistant Professor**

Kinesiology,

School of Public Health, Indiana University

**Allison Gruber, PhD, Assistant Professor**

Kinesiology

School of Public Health, Indiana University

**Zhongxue Chen, PhD, Associate Professor**

Epidemiology and Biostatistics

Indiana University

**Timothy Mickleborough, PhD, Professor**

Kinesiology

School of Public Health, Indiana University

**Sharlene Newman, PhD, Professor**

Psychological and Brain Sciences

Indiana University

1. **Background**

In the United States, every year approximately 1.7 million people sustain a traumatic brain injury (TBI) with the majority (80% or 1.4 millions) of these injuries falling into the category of concussion or mild TBI (mTBI).^1^ Concussion is classified as a mild form of traumatic brain injury, while changes in neural function following concussions are far from benign. Concussed athletes frequently experience lingering neurological symptoms, including headache, irritability, blurred vision, and confusion.^2^ In respect to these symptoms, researchers have utilized various metrics to study brain damage, including brain-derived blood biomarkers, sensory systems, and neuroimaging. However, due to exceedingly high intra-group variability in the concussed cohort, to date there is no gold standard metric to gauge severity of brain damage following head impacts.^3^

One possible explanation of the high variability is due to subconcussive head impact (SHI), defined as an impact to the head that does not cause any symptoms of concussion, regardless of magnitude of the impact. It, however, has the potential to cause insidious effects in the brain if sustained repetitively over time.^4^ An average athlete in contact sports experiences nearly a thousand subconcussive hits per season.^5^ While the vast majority of research has focused on a single impact that elicits concussion (e.g., disorientation, headache), many athletes are frequently exposed to subconcussive head impacts prior to the concussive blow.^6, 7^ Thus, it remains completely unknown whether neuronal damage and sensory deficit are caused by a single concussive blow, repetitive subconcussive impacts before the concussive blow, or both.

***Neurophysiological changes associated with SHI.*** Soccer headings are considered subconcussive head impacts, which are defined as head impacts that do not result in clinical symptoms of concussion.^4, 8^ However, recurring SHI can trigger axonal microtubule and neurofilament disruptions^9-11^ and blood-brain barrier impairment.^12^ Our previous clinical studies have revealed significant associations between the frequency/magnitude of SHI and ocular-motor impairment,^8, 13^ as well as neuronal damage reflected by increased levels of neural-injury blood biomarkers.^14, 15^ Specifically, we have observed that oculomotor nerve function is chronically impaired by 10 or more SHI from soccer heading and American football tackles.^16, 17^ These SHI also induced astrocyte activation/damage in the brain, as reflected by increased plasma levels of S100B,^15^ and induced axonal damage as measured by increased plasma levels of microtubule-derived neurofilament light polypeptide (NF-L) and tau proteins.^14^ Subsequent findings from neuroimaging studies have confirmed and extended our observations, by identifying the SHI effect to be more pronounced in brains that are not fully developed (until the mid-20s).^18^ For example, adolescent and young adult athletes aged 14-24 years who experience repetitive SHI have shown an axonal diffusion in white matter tracts of the limbic system,^19^ prefrontal cortex, longitudinal fasciculus,^20, 21^ and supramarginal gyrus,^22^ which are important for emotional stability, higher decision making, and language, respectively.

***Behavioral and cognitive consequences of SHI.*** Long-term, repetitive SHI is suggested as one of the strongest predictors for development of chronic traumatic encephalopathy (CTE).^23, 24^ This recently discovered progressive neurodegenerative pathology can result in neurocognitive decline as reflected by the occurrence of impulsive behaviors, depression, substance abuse, and suicidal ideation and attempts.^25^ If SHI-induced neural injuries continue, they can lead to increased risks of CTE as a function of an athlete’s career duration, and the level at which they play (i.e., professional, college).^23, 24, 26^ CTE has been identified in a number of former athletes that have engaged in contact sports (i.e., soccer, American football)^23, 27-29^ and in autopsies of 110 out of 111 brains (99%) of professional football players and 48 of 53 brains (91%) of college football players.^24^ The increased severity of CTE is also associated with a greater frequency of total SHI sustained during an athlete’s career. This is reflected in increased observations of violence, substance abuse, and suicide attempts.^24^

***Prevalence of SHI.*** Based on a survey conducted in 2006 by the Fédération Internationale de Football Association (FIFA), as many as 265 million people across all ages play soccer around the globe.^30^ In the U.S., participation rates for competitive collegiate men’s and women’s soccer have grown in the past decade: men, 18,512 in 2004 vs. 23,602 in 2014; women, 20,437 in 2004 vs. 26,358 in 2014.^31^ Soccer players maneuver their unprotected heads to deliberately impact the ball and direct it to pass, shoot, or clear.^32^ The average soccer player in college and professional settings experience a median frequency of 800 soccer headings (range: 50 to 2,100) in a season,^21, 33^ with an estimated total career (median of 12 years) of SHI exceeding 15,000 headers in some cases.^4, 34, 35^ Although athletes in American football, ice-hockey, and rugby wear protective gear (i.e., helmet, headband), they also experience neuronal damage from SHI because the protective gear *does not minimize brain displacement* within the skull.^36, 37^ For comparison purposes, a driver can experience 40 *g* of head and chest acceleration when a car collides into a fixed wall at 30 mph,^7, 8, 38^ whereby American football, ice-hockey, and rugby players can sustain an average magnitude of 22 to 32 *g* of head acceleration upon impact, with an average frequency of 650 SHI per single season.^4, 39-41^

***Soccer Heading Modality***

Soccer heading is a common skill performed by soccer athletes during practice and games. An average collegiate soccer players perform about 500 headers during a single season and over 3000 during the course of a career ^34, 42^  Research has demonstrated the need for increased awareness of potential brain injury associated with heading the soccer ball with some suggesting that the cumulative effect of soccer head impacts over a career may lead to outcomes similar to sustaining multiple concussions.^43^ Various studies have been conducted to examine the effect of an acute bout of soccer heading, including Dr. Kawata’s previous laboratory at Temple University, with most authors reporting no significant effect of heading on the brain function measures of neuropsychological or balance performance (Table 1). We have tested collegiate soccer players post heading ball speed up to 50 mph with no significant alterations in common clinical measures (e.g., balance error scoring system, signs and symptoms checklist). However, sensitive modalities like blood biomarker and ocular-motor testing begin to unravel subclinical perturbation caused by minor head hits if sustained repetitively.^16, 17, 44^

1. **Rationale and Specific Aims**

**Aim 1. Determine acute effects of subconcussive head impact on brain derived blood biomarkers using controlled soccer heading model.**

*Hypothesis***:** Repetitive subconcussive head impacts will significantly increase plasma levels of tau, GFAP, NF-L, UCH-L1, and S100B at 24h-post heading compared to baseline but not at 0h and 2h-post heading.

Outcome Variables: Primary outcome for this aim is group x time interaction on composite levels of all 5 markers at 24h post-intervention.

**Aim 2. Determine acute effects of subconcussive head impact on ocular-motor functions in a controlled soccer heading model.**

*Hypothesis:* Repetitive subconcussive head impacts will significantly decrease convergence, saccadic function, and smooth pursuit performance.

Outcome Variables: Primary outcome of this aim is the group by time interaction of KDT speed at 0h-post heading/kicking. The secondary outcome includes within and between group analysis on KDT speed at 2h and 24h-post heading/kicking and KDT error and NPC at all post time points.

**Aim 3. Test subconcussive effects on neurocognitive functions.**

*Hypothesis:* Ten bouts of subconcussive impacts will not induce decline in neurocognitive function.

**Exploratory Aim 1. Determine whether subjects with ADD/ADHD respond differently to subconcussive head impacts than those without ADD/ADHD.**

*Hypothesis:* Subjects with ADD/ADHD will perform significantly worse in behavioral, cognitive, and biochemical markers than those without ADD/ADHD.

1. **Inclusion/Exclusion Criteria**

**Inclusion Criteria**

For Soccer Cohort

1) being between 18 to 26 years of age

2) an active or former member of a soccer team (i.e., collegiate, intramural, club, professional)

3) at least 5 years of soccer heading experience.

**Exclusionary criteria**

For both Soccer and Non-Athletic Control cohorts

1) any head, neck, or face injury in the 1 year prior to the study (e.g., concussion, eye injury);

2) history of vestibular, ocular, or vision dysfunction (e.g., macular degeneration)

3) currently taking any medications affecting balance (e.g., antibiotics)

4) pregnancy

5) HIV

6) any neurological disorders (e.g., seizure disorders, closed head injuries with loss of consciousness greater than 15 minutes, CNS neoplasm, spinal cord injury/surgery, history of stroke)

7) hypertension, cardiac arrhythmia, or pulmonary disease

8) lower extremity injury that would prohibit normal walking

9) metal implants in the head

10) implantation of cochlear device, cardiac pacemaker, medical fusion device, intracardiac lines, or neurostimulator (e.g., DBS, epidural/subdural VNS)

11) history of severe injury to the bones, joints, or muscles in either arm

Session-specific exclusion criteria will include:
12) slept less than 4 hours before the 1st and 2nd test day
13) drank more than 3 alcoholic drinks or used recreational drugs 24 hours before the 1st and 2nd test day

14) drank more than 3 cups of coffee in an hour before test sessions
15) glasses are prohibited (contact lens are okay) for safety purpose for the heading intervention

1. **Enrollment/Randomization**

Potential participants will be recruited via listserv email to undergraduate students in the School of Public Health-Bloomington, and interested participants will contact (phone or email) and meet with the investigator to discuss the project and ask questions. For ADD/ADHD part of the study, the potential subjects will be recruited via listserv email and flyer. The informed consent and Concussion History & Health History Questionnaire will be given to the potential candidates. Participants who meet the inclusion criteria and are free of exclusionary factors will advance to the testing procedures.

We will use dice-based randomization method to randomize group assignments. Up on inclusion of the participants, based on dice roll they will be randomly assigned into one of three groups: Heading group (number of dice 1, 2, 3) or kicking group (number of dice 4, 5, 6).

1. **Study Procedures**

**Research Design**

We will use a repeated measures design. The independent variable will be time (pre-test, 0h post-test, 2h post-test, 24h post-test). The kicking-control group will be used to analyze any effects from bodily damage, exercise, and normal changes in dependent variables over time**.** The dependent variables assessed over time will include symptom scores, ocular-motor function, blood biomarker concentration, and cognitive function. Impact kinematics will also be assessed during soccer heading.

The study consists of 4 data collection time points in 2-day period. The 1st collection - pre-heading baseline-, followed by soccer heading intervention. The 2nd collection, 0h-post-heading time point (immediately after headings). The 3rd collection, 2h-post-heading time point (2h after headings). The 4th collection, 24h-post-heading time point (2nd day). This study design will enable to test outcome measures over 3 acute phases (Figure 1). The 1st day will last 4.5 hours and 2nd day will last 1.5 hours.

In order to address the potential residual effects of previous soccer-related injury or head trauma, we will recruit additional 20 age- and gender-matched healthy-non-athletic controls, against subjects in the soccer heading group to validate whether baseline values of each parameter are comparable to a healthy non-athletic cohort. However, regardless of whether there will be a difference in baseline between soccer players and healthy-controls, the purpose of the current study is to determine acute subconcussive head impact effects in the athletic population. Thus, the changes between soccer players and healthy controls will not terminate the study.

***Fig 1. Study design.***

*2x4 repeated measures design will be used to assess acute effect of subconcussive head impact.*

*Soccer Heading Protocol*

When a subject who suffice inclusion criteria and free of exclusion criteria agree to participate in the study, he/she will be randomly assigned into one of three groups (heading, kicking-control, standing-control). A standardized and reliable soccer heading protocol will be used for the experiment. A triaxial accelerometer (Triax Technologies) embedded in a head-band pocket and positioned directly below the external occipital protuberance (inion) to monitor linear and rotational head accelerations. A JUGS soccer machine will be used to simulate a soccer throw-in with a standardized ball speed of 25mph across all 3 groups. The ball speed is similar to when soccer players make a long throw-in from the sideline to mid-field. Soccer players frequently perform this maneuver during practice and game. Subjects will stand approximately 40ft away from the machine to perform either the heading or kicking (Fig 1). Participants in the heading group perform 10 standing headers with 1 header per minute, whereas kicking control group performs 10 kicks. The subjects in the heading and kicking groups will be instructed to direct the ball back toward the JUGS soccer machine in the air, while the subjects in the standing group will remain static. The kicking control group will aid to distinguish effects of subconcussive impact from exercise, bodily damage, and/or simply a daily variation of the outcome measures. The first phase of acute subconcussive effects will be measured at 0h-post heading, indicating immediate sensory perturbation and neuronal damage, followed by measurement of the second phase, 2h-post neural inflammatory response. Lastly, ocular-motor function coupled with cognitive function at 24h-post heading will be measured to assess the third phase of subconcussive effects.

*Symptom Checklist*the post-concussion symptom scale, as a subset of the Sports Concussion Assessment Tool 3,^3^ will be used as a method of assessing the presence and severity of symptoms. This paper-pencil symptom checklist consists of 22 symptoms with 7-point Likert scale per symptom to monitor subject’s well-being. Participants will be instructed to report their current symptoms truthfully by circling symptom scores. Symptom scores will be manually transferred into an Excel spreadsheet for the future analyses. A tester or transferer will be blinded in regard to the group assignments and one’s performance in other test parameters.

*Blood Biomarker Assessment*

The subject will be asked to lay on supine position with arm relaxed at 10-degree flexion. Antecubital vein blood draws will be performed each test session to help determine serum biomarker concentrations. A certified phlebotomist will thoroughly clean the antecubital fossa with an alcohol swab and draw 4 ml of whole blood into serum vacutainer tubes with a 21G butterfly needle. A Total of 16 ml blood for the study (4 ml x 4 time points). For non-athletic control subjects, 1 blood sample of 4 ml will be drawn. After the blood draw, the subject will use gauze to maintain direct pressure for 3 mins and Band-Aid will be provided. The whole blood will be centrifuged at 3,000 revolutions per minute for 20 minutes at 4°C after 40 mins of coagulation. The serum will then be divided and transferred into 1 mL cryovials and flash frozen and stored at -80°C in Exercise Biochemistry lab.

*Ocular-Motor Assessment*

The King-Devick Test (KDT) is designed to examine neuro-ophthalmologic functional integrity by performing a total of 145 saccades while rapidly reading numbers aloud to complete the test ^45^. The KDT will be administered on a tablet held by participants. The participants will be given one trial “demonstration” cards for practice followed by three different test cards. The tablet records the total amount of time it takes participants to complete all three cards. The participants will be instructed read aloud, left to right and top to bottom, a series of numbers on the test cards as fast as they can while refraining from using his/her fingers as a reading guide. As the cards progress, the difficulty increases with guiding lines disappearing and number becoming denser. The total time (in seconds) from 3 cards will serve as one of outcome variables.

For near point of convergence, the participants will be instructed to maintain gaze on a 14-point font size letter. The target moves down the length of the accommodative ruler toward the participants’ eyes at a rate of 1 to 2 cm/s. The NPC will be measured when eye malalignment is observed by the tester or when the participant verbally signals upon experiencing diplopia. NPC will be measured twice, and the mean value will be used for analyses.

The subject will then perform eye-movement task using the EYE-SYNC headset. This visual-tracking protocol has been replicated and validated in a number of concussion and sleep deprivation studies,^46-51^ however to our knowledge this study for the first time will unravel subconcussive effects. Prior to testing, a Snellen chart will be used to verify that the subject has a normal or corrected-to-normal vision (minimum 20/30). The subject will be seated in a normally lit room and stabilize the headset with two hands while the elbows placed on the desk. The visual stimulus will be presented using a 120-Hz frame rate LCD screen in the headset and binocular eye movements will be tracked by a single camera secured in the headset. The test stimulus consists of a red circular target, 0.5° diameter in a visual angle with a 0.2° black dot in the center. The target moves in a circular clockwise trajectory of 10° radius at 0.4 Hz, with the target speed corresponding to 25°/s. The entire testing sequence will last approximately 3 mins consisted of a calibration and 2 consecutive test runs. Calibration of the eye position is conducted by having the subject fixate on a target presented at eight locations on the circular path of the test stimulus and one additional fixation point at the center of the circular path. The fixation target was presented at these nine locations in a randomized order. Each of the two test runs consists of 6 cycles of circular movement corresponding to 15 s in duration per test run. The subject will be instructed as “follow the movement of the target as closely as possible.”

*Neurocognitive Assessments*

The Immediate Post-Concussion Assessment and Cognitive Testing (ImPACT) will be used to assess the effects of sleep deprivation and subconcussive head impacts on the neurocognitive function. The ImPACT has been incorporated in athletes, military servicemen, and civilian, yielding one of the highest validities among other computer-based testings [i.e., Automated Neuropsychological Assessment Metrics (ANAM) and Axon Sports].54 Although the computer-based testing does incur inevitable subjective factors (subjects’ mood, fatigue, and time of day), the ImPACT battery shows acceptable test-retest reliability.55 This particular tool helps to identify compromised neurocognitive functioning in different areas, including attention span, reaction time, problem solving, and working and verbal memory. By comparing subjects’ post-intervention scores to their baseline performance, declines in functioning can be identified. The ImPACT takes about 25 minutes to complete. Subjects will be seated in front of a 20-inch desktop, where stimuli are presented on the screen. Subjects will be instructed to “read the instruction and complete the tasks as quickly as you can”. Data will be stored in the secured system and extracted for analysis.

1. **Reporting of Adverse Events or Unanticipated Problems involving Risk to Participants or Others**

Reporting Adverse Events
Keisuke Kawata, Ph.D. (primary investigator) will serve as the data safety monitor. The data safety monitor will monitor adverse event data, oversee procedures designed to protect the privacy of subjects, and coordinate the reporting of the outcome of any investigation of an adverse event. In the event of an adverse event, Dr. Kawata will report and cooperate with the IRB in any necessary investigation.

Adverse Events Associated with Soccer Heading

There is a risk of inducing symptoms such as headache, blurred vision, and disorientation during soccer heading testing, however ten soccer headers with mild head acceleration are commonly incorporated in soccer practice, and certainly soccer players utilize heading skill to direct a soccer ball for passing and scoring during practice and game. An average soccer player performs 500 headers over an entire season.^34, 42^ It is extremely unlikely to elicit concussion-like symptoms (headache, nausea, disorientation, blurred vision) from headings with mild head acceleration. The study protocol has been replicated in multiple laboratories, producing invaluable data to progress traumatic brain injury research. Moreover, our preliminary study in football players have shown that a player sustained 39 head hits with the total magnitude of 1,200*g* during a single practice, but with no change in symptoms.^16^ In order to minimize the risk of adverse events from soccer heading, we will only include soccer players who regularly play soccer and perform soccer headings.

Adverse Events Associated with Blood Draw

Complications of antecubital venous blood draws include bruising, local infection, phlebitis and injury to structures near the cubital vein. All of these complications are uncommon. To minimize these risks, certified phlebotomists (Keisuke Kawata, Megan Huibregtse, Maddie Nowak) will perform the blood draws using single-use needles, tube holders and test tubes. The skin of the antecubital region will be cleaned with alcohol to further reduce the risks of infections. Following each blood draw the participant will remain seated while maintaining direct pressure against the site of the needle insertion. After each blood draw, the participant will be given a Band-aid to cover the puncture site. When the participant returns for each test session the certified phlebotomist will check the puncture sites for any signs of complications (which would be referred for an additional medical examination).

Following the 48 hour post-test, the participant will be reminded to contact the research staff if they have any pain or discomfort associated with the procedure. Pain and dizziness/fainting are other potential risks associated with antecubital venous blood draws. During the blood collection, the participants will be placed in a supine position. Prior to sitting up the certified phlebotomist will talk to the participant to confirm that the individual is comfortable. Once the participant is in a seated position they will sit for a few minutes to ensure that they are not feeling dizzy or faint. Ice will be offered at the end of each testing session to minimize any pain.

Adverse Events Associated with Ocular-motor testing

There is no risk associated with King-Devick test and Near point of convergence. During performing ocular-motor tasks, participants may experience a transient headache and dizziness. Because the ocular-motor testing runs for 15 seconds and repeated twice, we believe that it is rare to elicit symptoms in the short period of testing. Based on the large-scale normative data (n=50,000) acquired by SynkThink Inc. among sports athletes and military servicemen, no one has claimed abnormal symptom due to the ocular-motor testing.

1. **Study Withdrawal/Discontinuation**

Data collection will require subjects to participate in 4 test sessions. Subjects may withdraw from the study at any time. A subject may also be withdrawn from the research without his/her consent if, for example, he/she 1) comes to the 1^st^ and/or 2^nd^ day with intoxication, 2) sustained injury prior to the 1^st^ and 2^nd^ day, 3) self-reported sleep deprivation for the 1^st^ and/or 2^nd^ day, 4) sustained a head injury prior to the 1^st^ and 2^nd^ day. If the subject completed for example entire 1^st^ day procedure (3 test sessions) and unable to participate in the 2^nd^ day (4^th^ test session), the subject will be reimbursed hourly for the 1^st^ day.

1. **Statistical Considerations**

Power analysis

According to our previous studies^16, 17, 52^ and preliminary findings, the effect size is estimated to be 0.8. Thirty subjects per group will estimate to yield a power of 0.90 with p<0.05 as significance level. Although we did not have any dropouts in aforementioned previous studies, based on the dropout rate, a total number of subject will be adjusted.

Data analysis

Data analysis will be conducted by Dr. Zhongxue Chen and Dr. Keisuke Ejima. They will be initially blinded to group assignments by simply given the data set indicating Group (A, B). However, for our statistical model of longitudinal assessment, time points (pre, 0h-post, 2h-post, 24h-post) will be revealed to Dr. Chen and Ejima. Our interest is to identify the changes in biomarker expression, ocular-motor, and neural function after subconcussive head impacts in the heading group; hence, each parameter at post-heading time points (0h, 2h, and 24h) will be compared with pre-heading baseline. Each time point of the control group (kicking) will aid to interpret whether or not changes in the heading group are due to subconcussive impact, exercise, bodily hit, or simply a daily variation. To this end, mixed-effect regression model (MRM) with random intercept will be used for all aims. The primary predictors (fixed effect) will be groups (heading and kicking), time of measurement (pre, 0h, 2h, 24h post heading/kicking), and the group by time interaction. We treat time as a categorical variable by providing dummy variables for each time and participants as a random effect. The model accounts for the repeated measures from the same participants and included potential covariates such as sex, age, body mass index, years of soccer heading experience, and number of concussion history. We will employ a Jackknife estimation (leave-one-out) to provide 95% confidence interval (CIs) for each estimated outcome value. All analyses were conducted using statistical software R (version 3.4.1) with package “nlme.” Significance level for the primary outcome/analysis is set as a two-tailed p value of 0.05.


**Study Endpoint**

Throughout the study, we set the study endpoint based on subjects’ well-being measured via verbal claim to researchers. Because the mild nature of head impact, we expect to observe a subtle change in our sensitive outcome variables after soccer headers. To date, there is virtually no scientific evidence available to set study endpoints using ocular-motor, blood biomarker, and neuronal activity, we will guide the study based on symptoms, meaning that if a subject experiences any concussion-related symptoms following blood draw, soccer heading, ocular-motor testing, cognitive testing, his or her participation will be terminated.

1. **Privacy/Confidentiality Issues**

All participant information, and even the fact that an individual is in the study, is considered confidential. Confidentiality will be assured in this study through several mechanisms. During interviews and treatments, the investigators and study coordinator will ensure physical privacy by conducting interviews in a closed room. Subjects will be assigned a subject number to help make data anonymous. The participant’s Protected Health Information will be used for research purposes only. No names will accompany any data that is used for publication. To reduce the risk of confidentiality loss, electric data collected during the study will be stored on the university server and data collection sheets will be stored in a locked file cabinet in a locked room. The data will be stored indefinitely for data quality purpose for potential investigation after publishing the data. Only study personnel will have access to the data. All members of the research team are certified through the CITI program. Individual subject results will not be shared with the participants or their agents. Data analysis and publication will not include any identifying information.

**Data Management**

For symptoms, blood biomarker, and ocular-motor function, we will utilize spreadsheet to organize data and store in the IU Box account. Microsoft excel does not have range-checks system, however when we run statistical analysis using Statistical Analysis System (SAS), which can identify outlier and appropriate range for acquired data.

*Symptom*

Symptom scores will be manually transferred into an Excel spreadsheet for the future analyses. A tester or transferer will be blinded in regards to the group assignments and one’s performance in other test parameters. The Excel spreadsheet will be saved in the university secured Box account, which will be backed up weekly.

*Ocular-motor function*

The eye-movement data acquired via the EYE-SYNC headset will be automatically saved in an Excel spreadsheet. The Excel spreadsheet will be saved in the university secured Box account, which will be backed up weekly. King-devick and near point of convergence will be recorded in a paper and transfer to Excel sheet.

*Blood Biomarker*
Biomarker expressions will be measured via Simoa HD-1 Analyzer and the data will be transferred into an Excel spreadsheet by the tester.

*Cognitive function*

Neurocognitive function will be measured via ImPACT test and the data will be transferred to an Excel spreadsheet by the tester.

1. **Follow-up and Record Retention**

Since we propose to include a total of 80 subjects in the study, we estimate data collection and primary analysis of 80 subjects will last approximately 15 months. The electric data collected during the study will be stored on the university server, and data collection sheets will be stored in a locked file cabinet in a locked room. The data will be stored indefinitely for data quality purpose for potential investigation after publishing the data.

**References**

1. Mondello S, Schmid K, Berger RP, et al. The challenge of mild traumatic brain injury: role of biochemical markers in diagnosis of brain damage. Medicinal research reviews 2014;34:503-531.

2. Collins MW, Grindel SH, Lovell MR, et al. Relationship between concussion and neuropsychological performance in college football players. Jama 1999;282:964-970.

3. McCrory P, Meeuwisse W, Aubry M, et al. Consensus statement on Concussion in Sport--the 4th International Conference on Concussion in Sport held in Zurich, November 2012. J Sci Med Sport 2013;16:178-189.

4. Bailes JE, Petraglia AL, Omalu BI, Nauman E, Talavage T. Role of subconcussion in repetitive mild traumatic brain injury. Journal of neurosurgery 2013;119:1235-1245.

5. Broglio SP, Eckner JT, Martini D, Sosnoff JJ, Kutcher JS, Randolph C. Cumulative head impact burden in high school football. Journal of neurotrauma 2011;28:2069-2078.

6. Beckwith JG, Greenwald RM, Chu JJ, et al. Timing of concussion diagnosis is related to head impact exposure prior to injury. Med Sci Sports Exerc 2013;45:747-754.

7. Beckwith JG, Greenwald RM, Chu JJ, et al. Head impact exposure sustained by football players on days of diagnosed concussion. Med Sci Sports Exerc 2013;45:737-746.

8. Bokuda K, Shimizu T, Kimura H, et al. Quantitative analysis of the features of fasciculation potentials and their relation with muscle strength in amyotrophic lateral sclerosis. Neurol Sci 2016.

9. Alosco ML, Tripodis Y, Jarnagin J, et al. Repetitive head impact exposure and later-life plasma total tau in former National Football League players. Alzheimers Dement (Amst) 2017;7:33-40.

10. Ahmadzadeh H, Smith DH, Shenoy VB. Viscoelasticity of tau proteins leads to strain rate-dependent breaking of microtubules during axonal stretch injury: predictions from a mathematical model. Biophys J 2014;106:1123-1133.

11. Yap YC, King AE, Guijt RM, et al. Mild and repetitive very mild axonal stretch injury triggers cystoskeletal mislocalization and growth cone collapse. PloS one 2017;12:e0176997.

12. Marchi N, Bazarian JJ, Puvenna V, et al. Consequences of repeated blood-brain barrier disruption in football players. PloS one 2013;8:e56805.

13. Igarashi K, Hotta K, Imai K, et al. Can Positron Emission Tomography Detect Colorectal Adenomas and Cancers? J Gastroenterol Hepatol 2016.

14. Kawata K, Mitsuhashi M, Aldret R. A preliminary report on brain-derived extracellular vesicle as unique blood biomarkers for sport-related concussions. Frontiers in neurology 2018;9:239.

15. Kawata K, Rubin LH, Takahagi M, et al. Subconcussive Impact-Dependent Increase in Plasma S100beta Levels in Collegiate Football Players. J Neurotrauma 2017;34:2254-2260.

16. Kawata K, Rubin LH, Lee JH, et al. Association of Football Subconcussive Head Impacts With Ocular Near Point of Convergence. JAMA Ophthalmol 2016;134:763-769.

17. Kawata K, Tierney R, Phillips J, Jeka JJ. Effect of Repetitive Sub-concussive Head Impacts on Ocular Near Point of Convergence. Int J Sports Med 2016;37:405-410.

18. Johnson SB, Blum RW, Giedd JN. Adolescent maturity and the brain: the promise and pitfalls of neuroscience research in adolescent health policy. J Adolesc Health 2009;45:216-221.

19. Kuzminski SJ, Clark MD, Fraser MA, et al. White Matter Changes Related to Subconcussive Impact Frequency during a Single Season of High School Football. AJNR American journal of neuroradiology 2018;39:245-251.

20. Bahrami N, Sharma D, Rosenthal S, et al. Subconcussive Head Impact Exposure and White Matter Tract Changes over a Single Season of Youth Football. Radiology 2016;281:919-926.

21. Lipton ML, Kim N, Zimmerman ME, et al. Soccer heading is associated with white matter microstructural and cognitive abnormalities. Radiology 2013;268:850-857.

22. Slobounov SM, Walter A, Breiter HC, et al. The effect of repetitive subconcussive collisions on brain integrity in collegiate football players over a single football season: A multi-modal neuroimaging study. Neuroimage Clin 2017;14:708-718.

23. McKee AC, Stern RA, Nowinski CJ, et al. The spectrum of disease in chronic traumatic encephalopathy. Brain : a journal of neurology 2013;136:43-64.

24. Mez J, Daneshvar DH, Kiernan PT, et al. Clinicopathological Evaluation of Chronic Traumatic Encephalopathy in Players of American Football. Jama 2017;318:360-370.

25. Baugh CM, Robbins CA, Stern RA, McKee AC. Current understanding of chronic traumatic encephalopathy. Curr Treat Options Neurol 2014;16:306.

26. Goldstein LE, Fisher AM, Tagge CA, et al. Chronic traumatic encephalopathy in blast-exposed military veterans and a blast neurotrauma mouse model. Science translational medicine 2012;4:134ra160.

27. Hales C, Neill S, Gearing M, Cooper D, Glass J, Lah J. Late-stage CTE pathology in a retired soccer player with dementia. Neurology 2014;83:2307-2309.

28. Ling H, Morris HR, Neal JW, et al. Mixed pathologies including chronic traumatic encephalopathy account for dementia in retired association football (soccer) players. Acta neuropathologica 2017;133:337-352.

29. Grinberg LT, Anghinah R, Nascimento CF, et al. Chronic Traumatic Encephalopathy Presenting as Alzheimer's Disease in a Retired Soccer Player. Journal of Alzheimer's disease : JAD 2016;54:169-174.

30. FIFA Communications Division. FIFA Big Count 2006: 270 million people active in football [online]. Available at: <https://www.fifa.com/mm/document/fifafacts/bcoffsurv/bigcount.statspackage_7024.pdf>.

31. Irick E. Student-athlete participaiton: 1981-1982 to 2013-2014 [online]. Available at: <https://www.ncaa.org/sites/default/files/Participation%20Rates%20Final.pdf>. Accessed Nov 10.

32. Kirkendall DT, Garrett WE, Jr. Heading in Soccer: Integral Skill or Grounds for Cognitive Dysfunction? Journal of athletic training 2001;36:328-333.

33. Spiotta AM, Bartsch AJ, Benzel EC. Heading in soccer: dangerous play? Neurosurgery 2012;70:1-11; discussion 11.

34. Matser EJ, Kessels AG, Lezak MD, Jordan BD, Troost J. Neuropsychological impairment in amateur soccer players. Jama 1999;282:971-973.

35. Matser JT, Kessels AG, Jordan BD, Lezak MD, Troost J. Chronic traumatic brain injury in professional soccer players. Neurology 1998;51:791-796.

36. Pellman EJ, Viano DC, Withnall C, Shewchenko N, Bir CA, Halstead PD. Concussion in professional football: helmet testing to assess impact performance--part 11. Neurosurgery 2006;58:78-96; discussion 78-96.

37. Lloyd J, Conidi F. Comparison Of Common Football Helmets In Preventing Concussion, Hemorrhage And Skull Fracture Using A Modified Drop Test. Neurology 2014;82:P5.320.

38. King D, Hume PA, Brughelli M, Gissane C. Instrumented mouthguard acceleration analyses for head impacts in amateur rugby union players over a season of matches. Am J Sports Med 2015;43:614-624.

39. Guskiewicz KM, Mihalik JP, Shankar V, et al. Measurement of head impacts in collegiate football players: relationship between head impact biomechanics and acute clinical outcome after concussion. Neurosurgery 2007;61:1244-1252; discussion 1252-1243.

40. Schnebel B, Gwin JT, Anderson S, Gatlin R. In vivo study of head impacts in football: a comparison of National Collegiate Athletic Association Division I versus high school impacts. Neurosurgery 2007;60:490-495; discussion 495-496.

41. Crisco JJ, Fiore R, Beckwith JG, et al. Frequency and location of head impact exposures in individual collegiate football players. J Athl Train 2010;45:549-559.

42. Kaminski TW, Wikstrom AM, Gutierrez GM, Glutting JJ. Purposeful heading during a season does not influence cognitive function or balance in female soccer players. Journal of clinical and experimental neuropsychology 2007;29:742-751.

43. Jordan SE, Green GA, Galanty HL, Mandelbaum BR, Jabour BA. Acute and chronic brain injury in United States National Team soccer players. The American journal of sports medicine 1996;24:205-210.

44. Hwang S, Ma L, Kawata K, Tierney R, Jeka JJ. Vestibular Dysfunction after Subconcussive Head Impact. J Neurotrauma 2016.

45. Rizzo JR, Hudson TE, Dai W, et al. Objectifying eye movements during rapid number naming: Methodology for assessment of normative data for the King-Devick test. J Neurol Sci 2016;362:232-239.

46. Maruta J, Ghajar J. Detecting eye movement abnormalities from concussion. Progress in neurological surgery 2014;28:226-233.

47. Maruta J, Heaton KJ, Kryskow EM, Maule AL, Ghajar J. Dynamic visuomotor synchronization: quantification of predictive timing. Behav Res Methods 2013;45:289-300.

48. Maruta J, Lee SW, Jacobs EF, Ghajar J. A unified science of concussion. Annals of the New York Academy of Sciences 2010;1208:58-66.

49. Maruta J, MacDougall HG, Simpson JI, Raphan T, Cohen B. Eye velocity asymmetry, ocular orientation, and convergence induced by angular rotation in the rabbit. Vision research 2006;46:961-969.

50. Maruta J, Suh M, Niogi SN, Mukherjee P, Ghajar J. Visual Tracking Synchronization as a Metric for Concussion Screening. Journal of Head Trauma Rehabilitation 2010;25:293-305.

51. Tong J, Maruta J, Heaton KJ, et al. Degradation of Binocular Coordination during Sleep Deprivation. Frontiers in neurology 2016;7:90.

52. Zonner SW, Ejima K, Fulgar CC, et al. Oculomotor response to cumulative subconcussive head impacts in high school football players: a pilot longitudinal study. JAMA Ophthalmol 2019;137:265-270.
